# The distribution of IQ index scores in the psychometric profile of children with High Intellectual Potential (HIP): Is the heterogeneity specific to HIP?

**DOI:** 10.1192/j.eurpsy.2023.1568

**Published:** 2023-07-19

**Authors:** S. Hamdioui, L. Vaivre-Douret

**Affiliations:** 1 Department of Psychology, Faculty of Society and Humanity, University of Paris Cité, Paris; 2 INSERM Unit 1018-CESP, Faculty of Medicine, University of Paris-Saclay, UVSQ, Villejuif; 3 Faculty of Health, Department of Medicine Paris Descartes, University of Paris Cité; 4 Chair in Clinical Neurodeveloppmental Phenotyping, Institut Universitaire de France (IUF); 5 Department of Endocrinology, IMAGINE Institute of Necker-Enfants Malades hospital; 6 Department of Child Psychiatry, Necker-Enfants Malades Hospital, AP-HP.Centre, Paris, France

## Abstract

**Introduction:**

The majority of studies on the HIP IQ attest a heterogeneity of the IQ profile as specific to HIP. However, the samples are recruited in clinical consultations. Thus, it is important to investigate new samples from schools.

**Objectives:**

We aimed to analyze the index scores of the IQ profile of children without disorders or specific school assistance.

**Methods:**

The WISC-V was conducted and analyzed in 80 healthy children (50 HIP vs. 30 non-HIP), aged 7-to-13 years-old (mean 10y; SD 1.8). All children were recruited in private and public schools in Paris.

**Results:**

All IQ index scores were significantly higher in the HIP vs. non-HIP. In both groups, the Verbal Comprehension Index was the highest index while the Processing Speed Index was the lowest. There are significantly (p=0.02) more heterogeneous IQ profiles in HIP (64%) vs. non-HIP (47%), with a significantly larger gap between the highest and lowest index (respectively: median = 29.5 vs. 21.5). There was a significant-positive correlation between IQ level in general and the heterogeneity of the profile (r = 0.42; p<0.001).

**Conclusions:**

The HIP children show better verbal, visual-spatial, fluid reasoning, working memory, and processing speed index scores. However, the distribution of IQ index scores was similar in both groups. Thus, the heterogeneity of the IQ profile is not specific to HIP children, but rather related to IQ score level. This highlights the importance of considering the IQ as a continuum rather than as a categorical distribution. Moreover, it points to the interest to better understand the IQ profile by completing it with multidimensional assessments.

**Disclosure of Interest:**

None Declared

